# Significant Improvement Selected Mediators of Inflammation in Phenotypes of Women with PCOS after Reduction and Low GI Diet

**DOI:** 10.1155/2017/5489523

**Published:** 2017-06-05

**Authors:** Małgorzata Szczuko, Marta Zapałowska-Chwyć, Dominika Maciejewska, Arleta Drozd, Andrzej Starczewski, Ewa Stachowska

**Affiliations:** ^1^Department of Biochemistry and Human Nutrition, Pomeranian Medical University, Szczecin, Poland; ^2^Clinic of Gynecology and Urogynecology, Pomeranian Medical University, Szczecin, Poland

## Abstract

Many researchers suggest an increased risk of atherosclerosis in women with polycystic ovary syndrome. In the available literature, there are no studies on the mediators of inflammation in women with PCOS, especially after dietary intervention. Eicosanoids (HETE and HODE) were compared between the biochemical phenotypes of women with PCOS (normal and high androgens) and after the 3-month reduction diet. Eicosanoid profiles (9(*S*)-HODE, 13(*S*)-HODE, 5(*S*)-HETE, 12(*S*)-HETE, 15(*S*)-HETE, 5(*S*)-oxoETE, 16(*R*)-HETE, 16(*S*)-HETE and 5(*S*), 6(*R*)-lipoxin A_4_, 5(*S*), 6(*R*), 15(*R*)-lipoxin A_4_) were extracted from 0.5 ml of plasma using solid-phase extraction RP-18 SPE columns. The HPLC separations were performed on a 1260 liquid chromatograph. No significant differences were found in the concentration of analysed eicosanoids in phenotypes of women with PCOS. These women, however, have significantly lower concentration of inflammatory mediators than potentially healthy women from the control group. Dietary intervention leads to a significant (*p* < 0.01) increase in the synthesis of proinflammatory mediators, reaching similar levels as in the control group. The development of inflammatory reaction in both phenotypes of women with PCOS is similar. The pathways for synthesis of proinflammatory mediators in women with PCOS are dormant, but can be stimulated through a reduction diet. Three-month period of lifestyle change may be too short to stimulate the pathways inhibiting inflammatory process.

## 1. Introduction

The Rotterdam criteria in the diagnosis of polycystic ovary syndrome (PCOS) have been used since 2003, which led to the situation that such diagnosed women differ in phenotypes regarding disorders in androgen levels [1]. Women with PCOS are susceptible to numerous health problems related to the cumulation of adipose tissue, such as obesity, insulin resistance, type II diabetes, cardiovascular diseases, atherosclerosis, high blood pressure, endometrial cancer, infertility, and depression [2]. It seems that two phenotypes of women differ in mechanisms and intensity of pathological reactions which cause the disease [3]. Increased risk of the development of cardiovascular diseases and atherosclerosis in women with PCOS, especially with simultaneous high levels of androgens, lead us to study the differences between the phenotypes in metabolic pathways of eicosanoids. Nest stage was to observe women with PCOS regarding the changes undergoing in mediators of inflammation in response to reduction diet. In the available literature, there have been no studies on inflammatory mediators in PCOS, especially their changes after the diet.

The patients with PCOS are much more prone to estrogen-dependent tumours, as well as those of alimentary tract [4]. It is known that fatty acids affect carcinogenesis by, among others, the formation and metabolism of eicosanoids, that is, the products of the metabolism of fatty acids of high and various bioactivity. It happens through the changes in substrates availability and the effect on the activity of basic enzymes: cyclooxygenases and lipoxygenases [5]. Among the major mediators of inflammations are those formed by polyunsaturated fatty acids (PUFA), especially by arachidonic acid (AA) and linolenic acid (LA) [6, 7]. The mediators of hydroxyeicosatetraenoic acid are formed from AA through conversion by lipoxygenase [8]. The products of arachidonic acid (AA) oxidation, including prostaglandins (PGS), tolboxanes (TXS), hydroxyeicosatetraenoic acids (HETES), and hydroxyoctadecadienoic acids (HODES), are those which influence atherosclerosis pathogenesis [9]. Bojić et al. even postulate that 13-HODE and 9-HODE should be regarded as atherosclerosis biomarkers [10]. Higher levels of 13-HODE were observed also in patients with hypertension [11].

Hydroxyoctadecadienoic acid (9-HODE, 13-HODE), synthetized from LA, plays an important role in inflammatory process through the regulation of monocyte and macrophage function. 9-HODE has strong proinflammatory properties, while 13-HODE affects higher absorption of lipids and reverses cholesterol transport via the mechanism involving PPAR and through enhancing apoptosis [12, 13]. At the same time, 13-HODE inhibits the proinflammatory properties of 9-HODE [14–16].

5-Hydroxyeicosatetraenoic acid and 5-oxoETE are known as strong inflammatory mediators and act in a similar way as those described above. 5-oxoETE is a strong chemoattractant for eosinophils, neutrophils, basophils, and monocytes. It stimulates the development of tumour cells and blocks the induction of apoptosis via the inhibition of 5-LOX. It plays a pathophysiological role in asthma, allergy, tumour development, and cardiovascular diseases [17].

12-HETE has numerous biological properties. It is a strong proinflammatory chemoattractant for neutrophils. Moreover, 12-HETE affects endothelial cell adhesivity and the reorganisation of cytoskeleton and the production of cytokines [18].

15-Hydroxyeicosatetraenoic acid plays an important role in inflammation processes in such diseases as asthma, arthritis, psoriasis, and dermatitis. Moreover, it stimulates mitogenesis of endothelial cells, increases the activity of granulocytes and lymphocytes, and stimulates the production of leukotrienes via 5-LOX [19].

Additionally, lipoxins originating from AA and resolvins coming from EPA, DHA, and DPA-ɷ6, as well as maresins, being derivatives of DHA, are the mediators inhibiting inflammatory reaction (proresolving mediators) [6]. The discovery of AA metabolic pathways synthetizing lipoxins via ASA-COX2, 15-LOX, and 5-HETE leads to the change in the common belief that the derivatives of polyunsaturated omega-6 fatty acid AA are the mediators of the inflammation. Lipoxins definitely do not belong to this category [20]. The major proresolving functions of lipoxins are reduction of neutrophil transmigration, suppression of the release of proinflammatory cytokines by T-cells, and stimulation of phagocytic activity of monocytes and macrophages [6]. Other proinflammatory and proresolving factors were not studied here, pending the results of this study.

## 2. Material and Methods

### 2.1. Test Group (PCOS-I)

Diagnostic screening tests for PCOS were performed on 36 women at the age of 17–38 (26.76 ± 5.08) in the Clinic of Gynecology and Urogynecology, Pomeranian Medical University. The Ethics Committee of the Bioethical Commission of the Pomeranian Medical University in Szczecin gave approval for the study (No. KB-0012/134/12, with the addendum to the permission No. KB-0012/36/14). All the patients gave written informed consent, and their confidentiality and anonymity were protected. Rotterdam criteria were applied in PCOS diagnosis using USG (Ultrasound Voluson 730, GE, Switzerland). The test group comprised of Caucasian women aged 26.31 ± 5.52, with the average body weight 80.98 ± 16.06 kg and height 1.67 ± 0.06 m.

### 2.2. Classification into Phenotypes (NT and HT)

Women with PCOS have been divided into groups according to their testosterone level and the overall free androgen index (FAI). All results were compared between 3 groups: control group—normally ovulating women with proper BMI (CG), PCO high testosterone and/or high FAI (HT), PCO normal levels of testosterone (NT). Free androgen index (FAI) was calculated from the formula: FAI = 100 × (total testosterone nmol/l/SHBGnmol/l).

Electrochemiluminescence immunoassay (ECLIA method) was used to determine the levels of testosterone. ELISA method was used for quantitative determination of the concentration of androstenedione (Kobas Rosch E411).

### 2.3. Test Group after Dietary Intervention (PCOS-II)

After the three months of using the diet, 3 women were excluded from the test group due to pregnancy, 7 women did not appear for the control examination, and two women did not use the diet but only the recommendations. Finally, the test group comprised of 24 women with PCOS.

All participants of the study were characterized by low physical activity, at the level of the index PAL = 1.3–1.4. One of the recommendations provided with the diet was to increase the physical activity to at least 3 hours a week.

The average reduction of the body mass after three-month dietary intervention was 5.93 ± 2.53 kg.

### 2.4. Dietary Intervention

Before the study, every participant gave a written consent to take part in the research. Each woman received a seven-day menu adjusted to individual caloric requirements with the recommendations regarding the change of lifestyle. The compositions of the diets were calculated using nutrition software recommended by the National Food and Nutrition Institute (NFNI). In each case, the diet caloricity was reduced by about 600 kcal. The diet consisted of 5 meals per day. All products in the menu were specified by weight. Food products recommended in the diets were the sources of all macronutrients, in accordance with the food pyramid recommended by the NFNI (2016). The products used as the sources of carbohydrates (5 portions per day) were oatmeal, wholegrain rye bread or graham bread, brown rice, groats (wheat, millet, and buckwheat), sporadically potatoes, and wholemeal pasta. The carbohydrate products selected for the diets were characterized by lowered glycemic index (GI).

The following products were recommended in the diets as the source of protein (1 portion of meat and 2 portions of dairy products per day): eggs, lean meat without skin (turkey, chicken), fish, mainly sea fish (sole, salmon, tuna), semi-skimmed pasteurized milk and dairy products (quark, natural yoghurt, and buttermilk with 2% of fat), nuts and seeds (almonds, pumpkin seeds, sunflower seeds, sesame seeds, and poppy seeds), and legumes (soy, red lentils, beans, and peas).

The products being the sources of fat (2 portions per day, on average) were raw oils (rapeseed oil, and olive oil), oily fruits, such as avocado, as well as nuts, fish, meat, and dairy products.

Fruits and vegetables with low GI were also included in the diets. They were present in every meal to supplement the diet with vitamins and minerals. The patients were recommended to use braising, roasting, cooking in water, and steaming as heat treatment techniques to prepare their food.

### 2.5. HPLC Analysis

#### 2.5.1. Chemicals and Reagents

All chemicals used for isolation and chromatography analysis as methanol, acetonitrile, HCL, ethyl acetate, and acetic acid were HPLC grade and were purchased from Sigma Aldrich (St Louis, MO, USA) or Merck. Double-distilled water was obtained from a Milli-Q Water System (Millipore, Billerica, MA, USA). Buffers used for HPLC analysis were filtered through 0.22 *μ*m nylon filters (Agilent). Standards of arachidonic acid derivatives (AAD): 5(*S*)-HETE (5S-hydroxy-6E,8Z,11Z,14Z-eicosatetraenoic acid), 5(*S*)-oxoETE (5-oxo-6E,8Z,11Z,14Z-eicosatetraenoic acid), 12(*S*)-HETE (12S-hydroxy-5Z,8Z,10E,14Z-eicosatetraenoic acid), 15(*S*)-HETE (15S-hydroxy-5Z,8Z,11Z,13E-eicosatetraenoic acid), 16(*R*)-HETE (16R-hydroxy-5Z,8Z,11Z,14Z-eicosatetraenoic acid), 16(*S*)-HETE (16S-hydroxy-5Z,8Z,11Z,14Z-eicosatetraenoic acid), and 5(S),6(R)-lipoxin-A4 (5S,6R,15S-trihydroxy-7E,9E,11Z,13E-eicosatetraenoic acid), 5(S),6(R)15(R)-lipoxin-A4 (5(S),6(R),15(R)-trihydroxy-7E,9E,11Z,13E-eicosatetraenoic acid) as well as linoleic acid derivatives (LAD): 9(*S*)-HODE (9S-hydroxy-10E,12Z-octadecadienoic acid), and 13(*S*)-HODE (13S-hydroxy-9Z,11E-octadecadienoic acid) and internal standard prostaglandin B_2_ (PGB_2_) were obtained from Cayman Chemicals (Ann Arbor, MI, USA).

### 2.5.2. Sample Preparation

Venous blood was collected from the patients after an overnight fasting. Whole blood was collected and placed in tubes containing ethylenediaminetetraacetic acid (EDTA) as anticoagulant. The blood was immediately placed on ice or in a refrigerator, and next the samples were centrifuged at 3500 rpm for 10 min at 4°C within max 2 h of collection. Plasma was stored at −80°C. Standard blood biochemical analyses were performed in the University Hospital Laboratory. 5(*S*), 6(*R*)-lipoxin A_4_, 5(*S*), 6(*R*), 15(*R*)-lipoxin A_4_, 5(*S*)-HETE, 5(*S*)-oxoETE, 12(*S*)-HETE, 15(*S*)-HETE, 16(*R*)/16(*S*)-HETE, 9(*S*)-HODE, and 13(*S*)-HODE were extracted from 0.5 ml of plasma using a solid-phase extraction RP-18 SPE columns (Agilent Technologies, Cheadle, UK) [21]. Recovery and sensitivity of method were described previously [22]. The total recovery for all sample extraction and processing steps (mean ± SD) was 46 ± 8%.

### 2.5.3. Instrumentation

The HPLC separations were performed using Agilent Technologies 1260 liquid chromatograph consisting of a degasser (model G1379B), a binary pump (model G1312B), a column oven (model G1316A), and a diode-array detector DAD VL+ (model G1315C). Samples were injected with an autosampler (model G1329B). Agilent ChemStation software (Agilent Technologies) was used for instrument control, data acquisition, and analysis. The separation was completed on a Thermo Scientific Hypersil BDS C18 column 100 × 4.6 mm 3 *μ*m (cat no. 28,103-104630). The temperature of the column oven was set at 25°C.

### 2.5.4. HPLC Operating Parameters

For HPLC, we used a gradient method where the mobile phase was composed of a mixture of solvent A (methanol/water/acetic acid, 50/50/0.1, *v*/*v*/*v*) and solvent B (methanol/water/acetic acid, 100/0/0.1, *v*/*v*/*v*). The content of buffer B in the mobile phase was 30% at 0.0 min of separation, increased linearly to 80% at 20 min, reached 98% between 20.1 and 23.9 min, and 30% between 24 and 28 min [21]. The flow rate was 1.0 ml/min. The volume of injected sample was 60 *μ*l. The diode-array detector (DAD) monitored peaks by adsorption at 235 nm for 9(*S*)-HODE, 13(*S*)-HODE, 5(*S*)-HETE, 12(*S*)-HETE, and 15(*S)*-HETE, at 280 nm for 5(*S*)-oxoETE and internal standard prostaglandin B_2_, at 210 nm for 16(*R*)-HETE and 16(*S*)-HETE (the latter two were eluted as one peak), and at 302 nm for 5(*S*), 6(*R*)-lipoxin A4, 5(*S*), 6(*R*), and 15(*R*)-lipoxin A_4_. The absorbance spectra of peaks were analysed to confirm the identification of analytes. The peak-area ratios of every compound to PGB_2_ were calculated and plotted against the concentration standards of the calibration standards. The calibration curves were calculated by the least squares linear regression method in a range of concentration of ten standards from to 0.02 to 0.2 ug/ml.

### 2.6. Statistical Analysis

Statistical analyses were performed with Statistica 12.0. Parametric tests were used because the distribution in most cases was normal (Shapiro-Wilk test). To see the difference between the parameters before and after the diet (PCOS-I and PCOS-II), a paired *t*-test was used. In order to check the differences between PCO-I and GK or PCOS-II and GK, an unpaired *t*-test was used; *p* < 0.05 and *p* < 0.01 were considered as statistically significant.

## 3. Results

**Figure 1:**
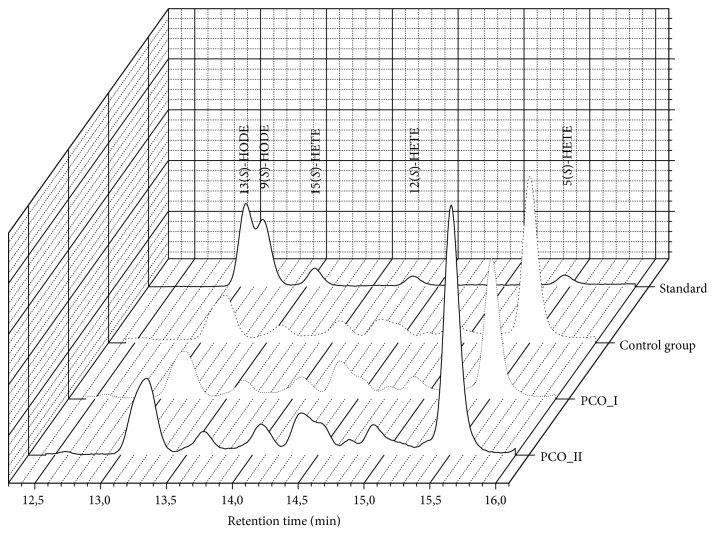
Chromatographic profile of samples of PCO-I, PCO-II, and control group corresponding to 0.1ug/ml of standards.

When comparing the concentrations of analysed inflammatory state mediators between the group of women with PCOS (PCOS-I) and the control group (CG), it was observed that statistically significant differences were present in levels of almost all eicosanoids except lipoxins A_4_ (Table 1, Figure 1). Similar relation with respect to the control group (CG) was observed when the patients were divided into two phenotypes: with high (HT) and normal (NT) levels of androgens. When we compared two phenotypes of women with PCOS (HT and NT) to each other, we did not observe any statistically significant differences. However, an increasing tendency was noted in the group of women with normal level of androgens (NT) (Table 1). After the three-month reduction diet with lower glycemic index, a significant increase in the majority of inflammatory factors was observed, except for 6(*R*), 15(*R*)-lipoxin A_4_ (Table 1). Women with PCOS (PCOS-II) after being three months on the diet did not differ significantly from the control group (CG), however, the levels of 12(*S*)-HETE were much higher (Table 1).

## 4. Discussion

**Figure 2:**
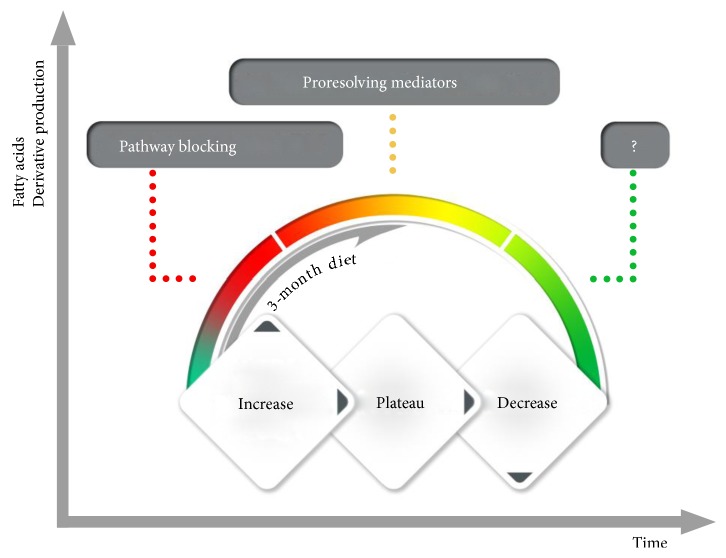
Synthesis of inflammatory reaction mediators in PCOS after dietary intervention.

This study revealed that the profile of lipid mediators may provide valuable information about PCOS. The increased expression of 5-LOX is associated with obesity, atherosclerosis, and insulin resistance [23]. It is the result of overexpression of activating protein in the adipose tissue with accompanying insulin resistance [24]. Its consequence is an increased synthesis of inflammatory mediators being the derivatives of arachidonic acid (AA) and linoleic acid (LA). Therefore, we assumed their higher levels in women with PCOS in comparison to potentially healthy women. On the contrary, it turned out that women with PCOS had lower levels of inflammatory mediators than women in the control group, which could be justified by the use of the mediators in repair reactions or by inefficient repair system, or due to chronic inflammatory process the mediators' synthesis pathways became dormant. As indicated by other authors, higher concentrations of 9-HODE, 13-HODE, and 5-HETE can be the result of increased oxidative stress [25]. However, in our study we did not observe increased concentrations of these derivatives, which, according to us, eliminates the effect of oxidative stress in PCOS pathogenesis [17]. It can only suggest that the system is ineffective or dormant. Our studies allow us to think that metabolic pathways for proinflammatory factors in both phenotypes of women with PCOS are not essentially different, but it seems that the synthesis of inflammatory mediators is higher in women with normal levels of androgens (NT). However, if we assume that the mechanism of the reaction is dormant, then women with high level of androgens (HT) are more susceptible to the damage of blood vessel walls and atherosclerosis. Repair mechanisms involve cell-mediated, humoral, hemostatic, and nervous and vascular responses—all of them are mutually related and decide on repair process being the essence of the inflammation. The accumulation of cholesterol, platelets, and immune system cells in the damaged site, and the activation of inflammatory process and the formation of xanthoma cells by accumulated macrophages, lead to the formation of atherosclerotic plaque [26]. Moreover, women with hypothyroidism, often present in patients with PCOS, are especially susceptible to arteriosclerosis. Both 9-HODE and 13-HODE were linearly and positively correlated with TSH, which confirms intensified arteriosclerotic processes [27]. HODE is formed from LDL via peroxidation of lipids as a marker of H+ donor activity [28]. 9-HODE is the strongest ligand participating, through GPR132, in the pathogenesis of arteriosclerosis [29]. On the other hand, HODES decrease the adhesion of platelets to endothelium wall. It is therefore hard to determine whether the effect of GPR132 on vascular cells is so negative [30]. Reduction diet used for three months by women with PCOS leads to the increase in the inflammatory mediators, that is, intensified the activity or activated the dormant repair processes. It is visible in analysed proinflammatory factors as well as, to a lesser extent, in lipoxins, being the resolving mediators inhibiting inflammatory reaction [20]. Thus, it seems probable that both in women with normal and high androgen levels the repair mechanisms were weakened (dormant), as compared to the control group, which could be caused by the diet low in MUFA and PUFA and rich in SFA. Only the properly balanced reduction diet resulted in reactivation of synthesis pathways and brought the repair processes to the level similar to that of the control group. Comparatively low concentration of lipoxins allow us to think that a 3-month period is too short for a diet for the majority of patients with PCOS. In our opinion, the pathways suppressing inflammatory process were not fully activated, but the peak of proinflammatory reaction has probably been reached. Our assumptions were illustrated in the figure below (Figure 2()). To strengthen the signal suppressing the inflammatory reaction after the patient with PCOS reached appropriate body weight, one should probably consider even higher intake of EPA and DHA and salicylic acid in the diet [31].

## 5. Conclusion

No significant differences were observed between the phenotypes of women with PCOS, differing in androgens levels, with respect to the synthesis of analysed inflammatory mediators (HETE, HODE, and lipoxins). Three-month reduction diet facilitates the processes of inflammatory mediators' synthesis, but is probably too short to assure the suppression of inflammatory reactions.

## Figures and Tables

**Table 1:** Eicosanoid concentration before (PCOS-I), after (PCOS-II) the three-month dietary intervention, and the control group (CG).

Eicosanoids	PCOS-I	CG	*p* value
	Mean [*μ*g/ml]	Mean [*μ*g/ml]	

6(*R*), 15(*R*)-lipoxin A_4_	0.26 ± 0.30	0.20 ± 0.16	NS
5(*S*), 6(*R*)-lipoxin A_4_	0.04 ± 0.10	0.05 ± 0.04	NS
16(*R*)/16(*S*)-HETE	**0.03** ± **0.04**	**0.08** ± **0.04**	*p* < 0.01
13(*S*)-HODE	**0.11** ± **0.08**	**0.21** ± **0.09**	*p* < 0.01
9(*S*)-HODE	**0.15** ± **0.11**	**0.24** ± **0.11**	*p* < 0.01
15(*S*)-HETE	**0.57** ± **0.45**	**1.01** ± **0.39**	*p* < 0.01
12(*S*)-HETE	**1.00** ± **1.02**	**1.72** ± **0.61**	*p* < 0.01
5(*S*)-oxoETE	**0.16** ± **0.20**	**0.47** ± **0.34**	*p* < 0.01
5(*S*)-HETE	**0.29** ± **0.25**	**0.69** ± **0.29**	*p* < 0.01

Eicosanoids	HT	CG	*p* value
	Mean [*μ*g/ml]	Mean [*μ*g/ml]	

6(*R*), 15(*R*)-lipoxin A_4_	0.27 ± 0.30	0.20 ± 0.15	NS
5(*S*), 6(*R*)-lipoxin A_4_	0.05 ± 0.13	0.05 ± 0.04	NS
16(*R*)/16(*S*)-HETE	**0.03 ± 0.04**	**0.08** ± **0.04**	*p* < 0.01
13(*S*)-HODE	**0.10 ± 0.08**	**0.21** ± **0.08**	*p* < 0.01
9(*S*)-HODE	**0.15 ± 0.12**	**0.24** ± **0.10**	*p* < 0.01
15(*S*)-HETE	**0.51 ± 0.37**	**1.01** ± **0.36**	*p* < 0.01
12(*S*)-HETE	0.85 ± 0.93	1.72 ± 0.57	NS
5(*S*)-oxoETE	**0.13 ± 0.14**	**0.47** ± **0.31**	*p* < 0.01
5(*S*)-HETE	**0.25 ± 0.22**	**0.69** ± **0.27**	*p* < 0.01

Eicosanoids	NT	CG	*p* value
	Mean [*μ*g/ml]	Mean [*μ*g/ml]	

6(*R*), 15(*R*)-lipoxin A_4_	0.26 ± 0.29	0.20 ± 0.15	NS
5(*S*), 6(*R*)-Lipoxin A_4_	0.03 ± 0.03	0.05 ± 0.04	NS
16(*R*)/16(*S*)-HETE	**0.04** ± **0.04**	**0.08** ± **0.04**	*p* < 0.01
13(*S*)-HODE	**0.12** ± **0.08**	**0.21** ± **0.08**	*p* < 0.01
9(*S*)-HODE	**0.15** ± **0.10**	**0.24** ± **0.10**	*p* < 0.01
15(*S*)-HETE	**0.67** ± **0.54**	**1.01** ± **0.36**	*p* < 0.01
12(*S*)-HETE	**1.24** ± **1.09**	**1.72** ± **0.57**	*p* < 0.01
5(*S*)-oxoETE	**0.22** ± **0.26**	**0.47** ± **0.31**	*p* < 0.01
5(*S*)-HETE	**0.35** ± **0.26**	**0.69** ± **0.27**	*p* < 0.01

Eicosanoids	HT	NT	*p* value
	Mean [*μ*g/ml]	Mean [*μ*g/ml]	

6(*R*), 15(*R*)-lipoxin A_4_	0.27 ± 0.30	0.26 ± 0.29	NS
5(*S*), 6(*R*)-lipoxin A_4_	0.05 ± 0.13	0.03 ± 0.03	NS
16(*R*)/16(*S*)-HETE	0.03 ± 0.04	0.04 ± 0.04	NS
13(*S*)-HODE	0.10 ± 0.08	0.12 ± 0.08	NS
9(*S*)-HODE	0.15 ± 0.12	0.15 ± 0.10	NS
15(*S*)-HETE	0.51 ± 0.37	0.67 ± 0.54	NS
12(*S*)-HETE	0.85 ± 0.93	1.24 ± 1.09	NS
5(*S*)-oxoETE	0.13 ± 0.14	0.22 ± 0.26	NS
5(*S*)-HETE	0.25 ± 0.22	0.35 ± 0.26	NS
Eicosanoids	PCOS-I	PCOS-II	*p* value
	Mean [*μ*g/ml]	Mean [*μ*g/ml]	

6(*R*), 15(*R*)-lipoxin A_4_	0.31 ± 0.31	0.23 ± 0.27	NS
5(*S*), 6(*R*)-lipoxin A_4_	0.03 ± 0.03	0.06 ± 0.08	NS
16(*R*)/16(*S*)-HETE	**0.04** ± **0.03**	**0.06** ± **0.04**	*p* < 0.01
13(*S*)-HODE	**0.13** ± **0.08**	**0.23** ± **0.08**	*p* < 0.01
9(*S*)-HODE	**0.16** ± **0.09**	**0.26** ± **0.10**	*p* < 0.01
15(*S*)-HETE	**0.75** ± **0.53**	**1.10** ± **0.46**	*p* < 0.01
12(*S*)-HETE	**1.45** ± **1.05**	**2.28** ± **1.43**	*p* < 0.01
5(*S*)-oxoETE	**0.25** ± **0.27**	**0.51** ± **0.34**	*p* < 0.01
5(*S*)-HETE	**0.38** ± **0.24**	**0.68** ± **0.28**	*p* < 0, 01

Eicosanoids	PCOS-II	CG	*p* value
	Mean [*μ*g/ml]	Mean [*μ*g/ml]	

6(*R*), 15(*R*)-lipoxin A_4_	0.23 ± 0.27	0.20 ± 0.16	NS
5(*S*), 6(*R*)-lipoxin A_4_	0.06 ± 0.08	0.05 ± 0.04	NS
16(*R*)/16(*S*)-HETE	0.06 ± 0.04	0.08 ± 0.04	NS
13(*S*)-HODE	0.23 ± 0.08	0.21 ± 0.09	NS
9(*S*)-HODE	0.26 ± 0.10	0.24 ± 0.11	NS
15(*S*)-HETE	1.10 ± 0.46	1.01 ± 0.39	NS
12(*S*)-HETE	2.28 ± 1.43	1.72 ± 0.61	NS
5(*S*)-oxoETE	0.51 ± 0.34	0.47 ± 0.34	NS
5(*S*)-HETE	0.68 ± 0.28	0.69 ± 0.29	NS

Values are expressed as mean ± SD, *p* < 0.01.

PCO-I: polycystic ovary syndrome, before dietetics intervention; PCO-II: polycystic ovary syndrome, after dietetics intervention; CG: control group; HT: high testosterone and/or high FAI; NT: normal levels of testosterone.
